# Monoaminergic levels at the forebrain and diencephalon signal for the occurrence of mutualistic and conspecific engagement in client reef fish

**DOI:** 10.1038/s41598-018-25513-6

**Published:** 2018-05-09

**Authors:** Murilo S. Abreu, João P. M. Messias, Per-Ove Thörnqvist, Svante Winberg, Marta C. Soares

**Affiliations:** 1 Graduation Program in Pharmacology, Federal University of Santa Maria (UFSM), Santa Maria, RS 97105-900 Brazil; 2 CIBIO, Centro de Investigação em Biodiversidade e Recursos Genéticos, Universidade do Porto, Campus Agrário de Vairão, 4485-661 Vairão, Portugal; 3 Department of Neuroscience, Uppsala University, Box 593, Husargatan 3, 75124 Uppsala, Sweden

## Abstract

Social interactions are commonly found among fish as in mammals and birds. While most animals interact socially with conspecifics some however are also frequently and repeatedly observed to interact with other species (i.e. mutualistic interactions). This is the case of the (so-called) fish clients that seek to be cleaned by other fish (the cleaners). Clients face an interesting challenge: they raise enough motivation to suspend their daily activities as to selectively visit and engage in interactions with cleaners. Here we aimed, for the first time, to investigate the region-specific brain monoaminergic level differences arising from individual client fish when facing a cleaner (interspecific context) compared to those introduced to another conspecific (socio-conspecific context). We show that monoaminergic activity differences occurring at two main brain regions, the diencephalon and the forebrain, are associated with fish clients’ social and mutualistic activities. Our results are the first demonstration that monoaminergic mechanisms underlie client fish mutualistic engagement with cleanerfish. These pathways should function as a pre-requisite for cleaning to occur, providing to clients the cognitive and physiological tools to seek to be cleaned.

## Introduction

The monoamines, serotonin (5-HT) and dopamine (DA), are essential neurotransmitters in the central nervous system. The vertebrate serotonergic system is characterized by well-defined nuclei, comprised of the raphe nuclei, and extends projections to the preoptic area and the basal hypothalamus^1–3^. In teleost fish, serotonergic fibers present similar projections, including the innervation of areas such as the telencephali, diencephalon, preoptic nucleus, pituitary gland, thalamus, and other regions^4^. The vertebrate dopaminergic system is comprised of projections that originate in the posterior tubercular *orthopedia*-dependent neurons, which include the individual somata integrating the ascending DA system, the descending diencephalospinal, as well as the endohypothalamic, circuitry^5^. These monoamines (5-HT and DA) are found in practically all brain regions^6,7^, and in individuals from all major species’ groups (e.g., mollusks^8,9^, teleost fish^10,11^ and humans^12,13^).

Monoamines are crucially involved in the orchestration of behaviour in vertebrates and invertebrates^14–17^, from simple to more complex social outputs that require coordination between two or more individuals^18,19^. 5-HT is best known to be responsible for the regulation of social motivation or mood in vertebrates, including humans^20–22^, however, variation in levels may also be associated with antisocial (impulsive) behaviours and aggressive responses^23,24^. DA is highly involved in the modulation of cognition^25,26^, decision making and reward processing^27,28^, as it is key to associative learning^29^. Moreover, effects of 5-HT on social behaviour have repeatedly been observed in fish. For instance, because zebrafish individuals form tight groups, isolation can be harmful; in cases of chronic social isolation (long-term social deprivation) these individuals seem to decrease serotonin levels^30^. Acute exposure to fluoxetine has also been demonstrated to decrease social interaction in zebrafish^31^. In addition, dyadic agonistic interactions in zebrafish have been shown to cause elevated brain serotonergic activity in subordinate zebrafish, as indicated by a rise of hindbrain serotonin ratios (5-hydroxyindolacetic acid (5-HIAA)/5-HT) ratios)^32^. However, knowledge concerning the role of monoaminergic action in specific brain regions during non-aggressive or non-sexual social behaviour, and most notably, cooperative behaviour between fish is still quite limited.

Social interactions are commonly found among fish, such as in mammals and birds. A classic example of fish sociality is the case of schooling behaviour, where fish need to coordinate position and movement within school to gain benefits related to predatory escape or foraging opportunities^33^. Most frequently, fish are observed to interact with other conspecifics during mating (male-female interactions) or in situations of between-male contests (aggressive behaviour)^34^. Notably, incidents of social interspecific interactions have also been reported, such as interspecific shoals^35^, events of foraging in mixed-species parties^36^, hunting with interspecific partners^37^ and finally, engaging in cooperative interactions (e.g., interspecific cleaning^38,39^). Indeed, during interspecific cleaning interactions, specialized fish known as cleaners receive the visit of other fish (known as clients), to inspect the body surface or gill chambers in search of ectoparasites, mucus and dead or diseased tissues^40–42^. One the best-known species of cleaner fish is the Indo-Pacific cleaner wrasse, *Labroides dimidiatus*, which undertakes interspecific cooperative interactions as means to secure energy. In this system, simple foraging has been replaced by a series of cognitive sophisticated behaviours that include: individual recognition of clients, manipulation of client decisions (on distinct levels of action), reconciliation, punishment, advertising of their cleaning services, tactical deception and indirect reciprocity based on image scoring (reviewed by^43^).

On the proximate level, these complex relationships are influenced by brain monoaminergic systems, with elevated serotonin causing increases in cleaner predisposition to interact and to provide more physical contact to clients (tactile stimulation)^44^. Interestingly, shifts in the perception of reward occur via blockage of dopamine receptors (D_1_ and D_2_ like), which induce cleaners to initiate more interactions and to provide greater amounts of physical contact to their partners^45,46^. The modulation of learning in the context of interspecific sociality seems also to be strongly associated with the increase in signaling of the dopamine D_1_ receptor^47,48^, and the attribution of motivational salience to cue-signals^49^.

Currently, our entire knowledge is derived from an approach to cleaners’ behaviour and monoaminergic response. From the clients’ perspective, studies have mostly focused on the benefits of reducing parasite infestation in relation to interrenal response levels and immune function^50,51^ and the effect of physical contact^52^. However, clients also face challenges: they must raise enough motivation to temporarily suspend their daily activities and go visit cleaners. Clients need to learn to seek, recognize and interact with cleaners, which will happen if they associate cleaner fish cue-signals with reward gain during their early life stages. Monoaminergic systems should be relevantly involved in both conspecific and/or interspecific (cooperative) relationships. However, the putative effects of different social contexts on serotonin and dopamine levels at different brain regions of clients remain to be discovered. Here we aimed to investigate the region-specific brain monoaminergic differences in individual client fish that are exposed to a cleaner (interspecific context), with others in contact with a conspecific (socio-conspecific context). We hypothesized that clients’ behavioural response to different social partners (experimental treatments) would associate with differences in brain 5-HT, DA levels and related metabolites.

## Results

### Client behaviour

There were differences in the behavioural response of clients (*Naso elegans*; see the Methods section for further details) between our two experimental treatments: client with cleaner (*L. dimidiatus*) and client with conspecific (*N. elegans*). Behavioural interactions occurred in both treatments, but not equally: clients interacted more with cleaners than with conspecifics (see Table 1). To further confirm whether the distinction between these two treatments was consistently expressed, we compared each behavioural measure. The frequency of cleaning interactions was significantly higher when clients were exposed to a cleaner compared to those exposed to another conspecific, and the same occurred with the mean interaction time, the frequency of cleaning bites, the proportion of interactions with tactile stimulation and the proportion of time providing tactile stimulation, except for the incidence of chases which showed the opposite trend (Table 1). For the frequency of client jolts, no difference between contacting with a cleaner or conspecific was found (Table 1).

**Table 1:** Observed behavioural measures for each experimental treatment.

Behaviour	Experimental treatments			
	Cleaner (n = 7)	Conspecific (n = 7)	Mann-Whitney U test	Cohen’s d
Number of interactions	5 ± 3.13	0	P = 0	1.38
Average interaction time (s)	1 ± 0.32	0	P = 0	1.84
Cleaning bites	5 ± 3.14	0 ± 0.7	P = 0.04	1.21
Proportion of interactions with tactile stimulation	0.4 ± 0.09	0 ± 0.02	P = 0.0	1.76
Proportion of time spent providing tactile stimulation	0.18 ± 0.19	0 ± 0.02	P = 0.02	0.9
Frequency of client jolts/100s	0 ± 0.3	0	P = 0.19	1.03
Frequency of chases	1 ± 0.53	21 ± 3.74	P = 0.01	2.12

Medians ± Standard Error (SEM) are provided for each behavioural measure.

### Client brain monoamines

**Figure 1:**
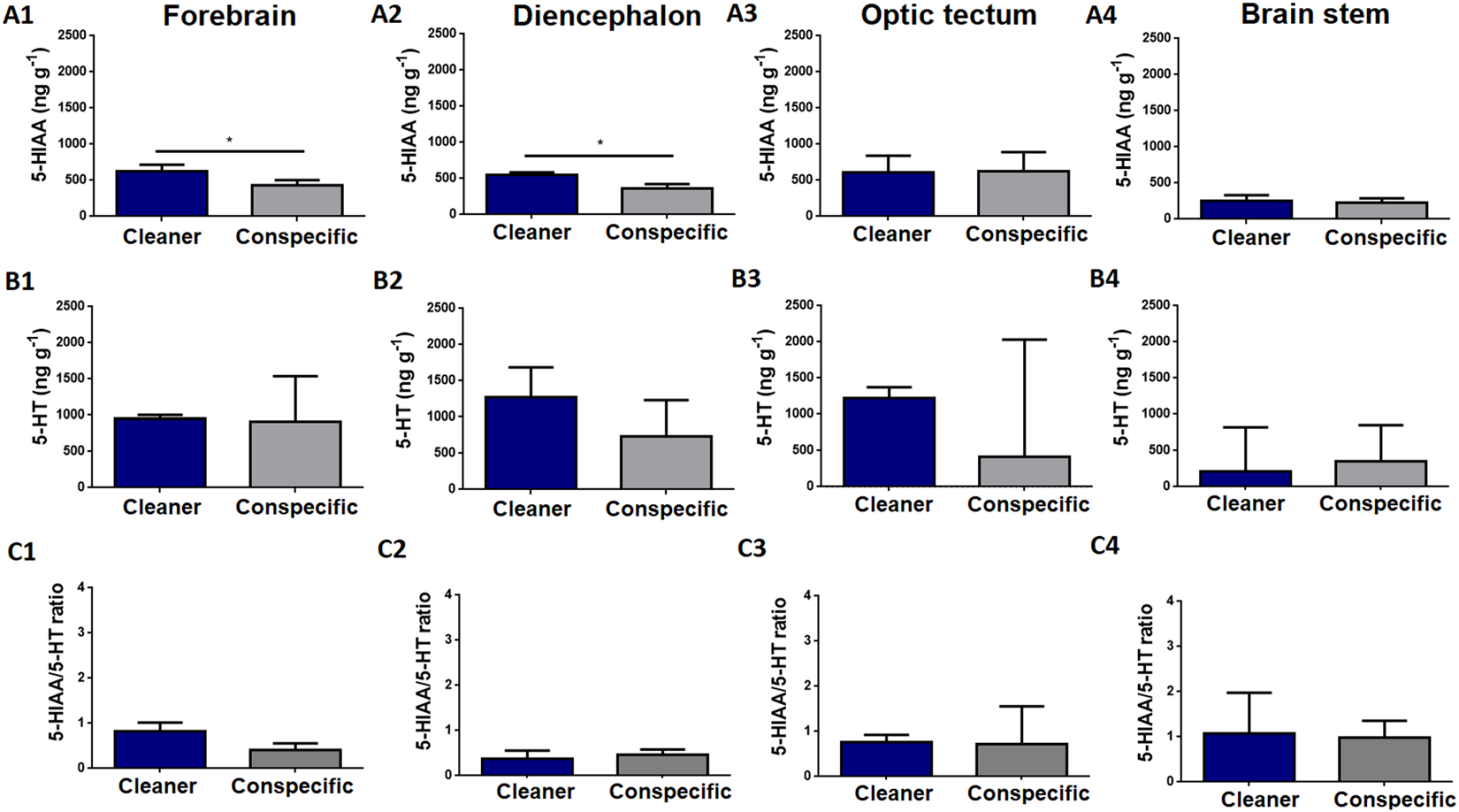
Serotonergic levels across clients’ brain areas exposed to a cleaner (*Labroides dimidiatus*) or a conspecific (*Naso elegans*). 5-hydroxyindoleacetic acid (5-HIAA) in the (A1) forebrain (FB) area (U = 8; p = 0.04; η2 = 0.32), (A2) diencephalon (DL) area (U = 6; p = 0.02; η2 = 0.27), (A3) optic tectum (OT) area (U = 23; p = 0.87; η2 = 0.01), (A4) brain stem (BS) area (U = 23; p = 0.87; η2 < 0.01). Serotonin (5-HT) in the (B1) FB area (U = 18; p = 0.45; η2 = 0.1), (B2) DL area (U = 16; p = 0.3; η2 = 0.12), (B3) OT area (U = 20; p = 0.6; η2 = 0.03), (B4) BS area (U = 16; p = 0.52; η2 = 0.01). 5-HIAA/5-HT ratio in the (C1) FB area (U = 11; p = 0.09; η2 = 0.29), (C2) DL area (U = 20; p = 0.6; η2 < 0.01), (C3) OT area (U = 23; p = 0.87; η2 = 0.01), (C4) BS area (U = 15; p = 0.43; η2 = 0.08). Medians and interquartile ranges are shown. Significant values from Mann-Whitney U tests are shown above bars: *<0.05.

Four brain regions were included in our analysis (see the Methods section for further details): forebrain (FB) (which included olfactory bulbs and telencephalon), diencephalon (DL), optic tectum (OT) and the brain stem (BS). Regarding 5-HIAA brain levels, clients exposed to a cleaner had higher 5-HIAA brain levels in the forebrain (Fig. 1 (A1)) and diencephalon (Fig. 1 (A2)), compared to clients in contact with a conspecific. No difference was found in 5-HIAA levels, 5-HT levels or 5-HIAA/5-HT ratios in any of the remaining brain regions analysed (Fig. 1).

**Figure 2:**
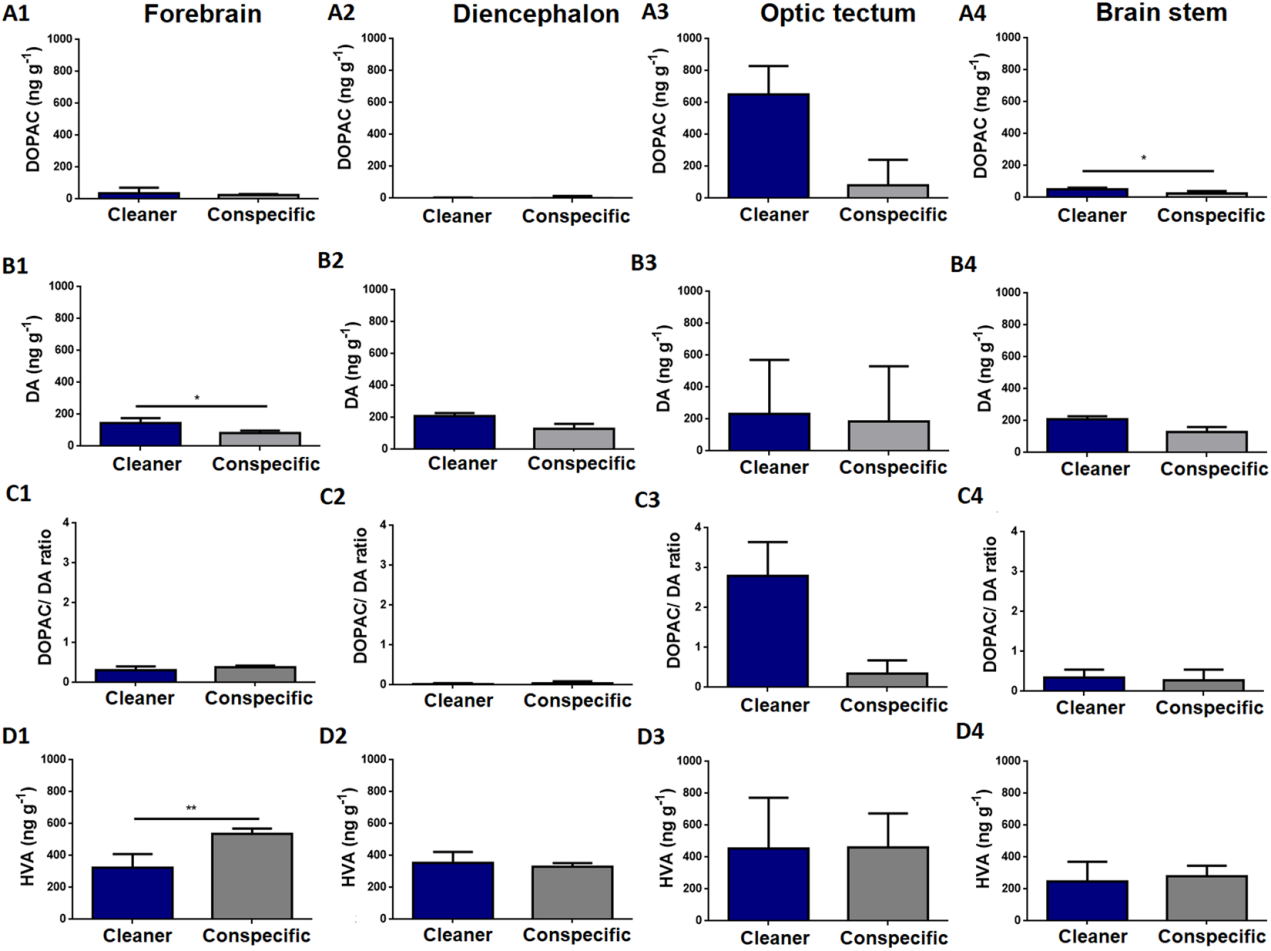
Dopaminergic and HVA levels across clients’ brain areas exposed to a cleaner (*Labroides dimidiatus*) or a conspecific (*Naso elegans*). 3,4-dihydroxyphenylacetic acid (DOPAC) in the (A1) forebrain (FB) area (U = 11; p = 0.1; η2 = 0.30), (A2) DL area (U = 11; p = 0.08; η2 = 0.2), (A3) optic tectum (OT) area (U = 10; p = 0.07; η2 = 0.37), (A4) brain stem (BS) area (U = 8; p = 0.04; η2 = 0.27). Dopamine (DA) in the (B1) FB area (U = 5; p = 0.01; η2 = 0.39), (B2) DL area (U = 9; p = 0.05; η2 = 0.3), (B3) OT area (U = 18; p = 0.45; η2 < 0.01), (B4) BS area (U = 14; p = 0.2; η2 = 0.13). DOPAC/DA ratio in the (C1) FB area (U = 15; p = 0.26; η2 = 0.05), (C2) DL area (U = 8; p = 0.57; η2 = 0.12), (C3) OT area (U = 15; p = 0.26; η2 = 0.28), (C4) BS area (U = 17; p = 0.38; η2 = 0.02). Homovanillic acid (HVA) in the (D1) FB area (U = 1; p < 0.01; η2 = 0.65), (D2) DL area (U = 13; p = 0.17; η2 = 0.17), (D3) OT area (U = 21; p = 0.69; η2 = 0.02), (D4) BS area (U = 20; p = 0.6; η2 = 0.01). Medians and interquartile ranges are shown. Significant values from Mann-Whitney U tests are shown above bars: *<0.05, **<0.01.

Clients exposed to a cleaner have higher DOPAC levels in the brain stem than clients in contact with a conspecific (Fig. 2 (A4)). In addition, clients exposed to a cleaner show higher DA concentrations in the forebrain compared to clients exposed to a conspecific (Fig. 2(B1)). On the other hand, clients in contact with a conspecific revealed to have higher homovanillic (HVA) tissue levels in the forebrain than clients introduced to a cleaner (Fig. 2 (D1)). Finally, treatment had no significant effect on: a) DA levels (in any other brain region), b) 3,4-dihydroxyphenylacetic acid (DOPAC) levels, c) HVA levels and d) DOPAC/DA ratios in all remaining brain regions analysed (Fig. 2). Finally, we analysed the relationships between clients’ behaviour and their brain 5-HIAA/5-HT and DOPAC/DA ratios in all four brain regions, however all revealed to be non-significant after p-value adjustments (Tables 2 and 3).

**Table 2:** Correlations (Spearman correlation coefficients) between each behavioural measure and serotonin levels in different brain macro-areas for the two experimental treatments: client exposed to a cleaner (*Labroides dimidiatus*) or a conspecific (*Naso elegans*).

Behaviour	Brain Macro-areas			
	Forebrain	Diencephalon	Optic Tectum	Brain Stem
Cleaner N = 7				
Number of interactions	r = −0.38	r = 0.49	r = −0.44	r = 0.44
	P = 0.35	P = 0.28	P = 0.28	P = 0.35
Average interaction time (s)	r = −0.27	r = 0.13	r = −0.27	r = 0.13
	P = 0.28	P > 0.99	P = 0.28	P > 0.99
Cleaning bites	r = −0.38	r = 0.49	r = −0.44	r = 0.44
	P = 0.35	P = 0.28	P = 0.28	P = 0.35
Proportion of interactions with tactile stimulation	r = 0.18	r = 0.36	r = 0.21	r = 0.46
	P = 0.71	P = 0.44	P = 0.66	P = 0.3
Proportion of time spent providing tactile stimulation	r = 0.18	r = 0.36	r = 0.21	r = 0.46
	P = 0.71	P = 0.44	P = 0.66	P = 0.3
Frequency of client jolts/100s	r = −0.35	r = 0.6	r = −0.6	r = 0.24
	P = 0.28	P = 0.17	P = 0.09	P = 0.63
Frequency of chases	r = −0.11	r = 0.5	r = −0.15	r = 0.69
	P = 0.7	P = 0.26	P = 0.64	P = 0.11
Conspecific N = 7				
Number of interactions	—	—	—	—
Average interaction time (s)	—	—	—	—
Cleaning bites	r = −0.53	r = 0.13	r = −0.44	r = −0.13
	P = 0.09	P = 0.8	P = 0.14	P = 0.6
Proportion of interactions with tactile stimulation	r = 0.4	r = −0.49	r = 0.75	r = 0.65
	P = 0.43	P = 0.09	P = 0.09	P = 0.33
Proportion of time spent providing tactile stimulation	r = 0.4	r = −0.49	r = 0.75	r = 0.65
	P = 0.43	P = 0.09	P = 0.09	P = 0.33
Frequency of client jolts/100s	—	—	—	—
Frequency of chases	r = −0.23	r = 0.36	r = 0.2	r = 0.38
	P = 0.58	P = 0.43	P = 0.67	P = 0.47

There were no statistically significant correlations.

**Table 3:** Correlations (Spearman correlation coefficients) between each behavioural measure and dopamine levels in different brain macro-areas for the two experimental treatments: client introduced to a cleaner (*Labroides dimidiatus*) or to a conspecific (*Naso elegans*).

Behaviour	Brain Macro-areas			
	Forebrain	Diencephalon	Optic Tectum	Brain Stem
Cleaner N = 7				
Number of interactions	r = −0.49	r = 0.06	r = 0.44	r = −0.22
	P = 0.23	P = 0.93	P = 0.35	P = 0.58
Average interaction time (s)	r = −0.4	r = 0	r = 0.67	r = −0.4
	P = 0.14	P = 0.57	P = 0.14	P = 0.14
Cleaning bites	r = −0.49	r = 0.06	r = 0.44	r = −0.22
	P = 0.23	P = 0.92	P = 0.35	P = 0.58
Proportion of interactions with tactile stimulation	r = −0.18	r = −0.08	r = 0.71	r = 0.32
	P = 0.71	P = 0.7	P = 0.09	P = 0.5
Proportion of time spent providing tactile stimulation	r = −0.18	r = −0.08	r = 0.71	r = 0.32
	P = 0.71	P = 0.7	P = 0.09	P = 0.5
Frequency of client jolts/100s	r = −0.66	r = −0.1	r = 0.42	r = −0.18
	P = 0.08	P = 0.28	P = 0.4	P = 0.55
Frequency of chases	r = −0.15	r = 0	r = 0.27	r = 0.23
	P = 0.64	P = 0.83	P = 0.56	P = 0.64
Conspecific N = 7				
Number of interactions	—	—	—	—
Average interaction time (s)	—	—	—	—
Cleaning bites	r = −0.49	r = 0.53	r = 0.44	r = −0.4
	P = 0.1	P = 0.24	P = 0.71	P = 0.14
Proportion of interactions with tactile stimulation	r = 0.13	r = −0.08	r = −0.27	r = 0.75
	P = 0.81	P = 0.48	P = 0.28	P = 0.09
Proportion of time spent providing tactile stimulation	r = 0.13	r = −0.08	r = −0.27	r = 0.75
	P = 0.81	P = 0.48	P = 0.28	P = 0.09
Frequency of client jolts/100s	—	—	—	—
Frequency of chases	r = −0.72	r = −0.32	r = 0.56	r = 0.04
	P = 0.07	P = 0.46	P = 0.2	P = 0.95

There were no statistically significant correlations.

## Discussion

In this study, we exposed clients to two different contexts: a conspecific and an interspecific situation. The results show that clients were mostly engaging in agonistic interactions when exposed to a conspecific, and mostly in cleaning interactions when kept with cleaners. However, interactions were most intense when clients were exposed to cleaners (see Table 1). We found 5-HIAA to be higher in the forebrain and diencephalon of clients exposed to cleaner, compared to those in a conspecific context. Regarding the dopaminergic system, DA was observed to be higher in the forebrain and DOPAC in the brain stem of fish exposed to a cleaner, compared to those in the conspecific situation; but showing an opposite tendency for forebrain HVA, with lower HVA levels in the clients exposed to a cleaner.

We observed that forebrain and diencephalic 5-HIAA concentrations were higher in clients interacting with a cleaner compared to clients interacting with a conspecific. Evidence is consistent with findings in other species of fish where the activation of the posterior tuberculum/hypothalamic 5-HT neuronal populations, and higher 5-HT activity, was positively correlated with overt aggression (i.e. bites) in fish introduced to a real opponent situation^53^. Higher serotonergic activity in the diencephalon has also been reported in fish following the loss of a fight^53^, while the event of winning a fight seems to contribute to a reduction of 5-HIAA/5-HT ratio in resident fish^54,55^. Considering that the 5-HIAA/5-HT ratio is mostly an indicator of neurotransmitter use, representing its release and metabolism^56–61^, this would mean that in an interspecific context, clients would have a higher serotonergic activity in the diencephalon, similar to what occurs with fish after the loss of a contest. Otherwise, the lower levels of 5-HIAA concentrations observed for clients exposed to a conspecific, may be associated with lower interrenal activity (i.e. lower cortisol levels), since stressed fish elevate serotonin levels at the hypothalamus, telencephalon and medulla oblongata^62^. Previous studies of the client’s perspective have focused on finding the physiological effects of interacting with cleaners, which have so far provided indications for stress reduction and immune benefits arising from client-cleaner interactions^50,51^. In fact, cleaners have not only been acknowledged to reduce client cortisol (stress) levels, by removing ectoparasites, but also by the simple provision of physical contact^52^. Thus, at this point, all evidence suggests that client-cleaner interactions can reduce client stress levels. Whether instances of poor cleaning service, i.e. bouts with lower amounts of physical stimulation, with lots of mucus gleaning and bites instead of parasite removal, will aggravate client mood and with its acute stress levels, is yet to be determined.

Similarly, clients in the interspecific treatment had significantly higher DA in the forebrain, which should mostly relate to cleaning engagement. The fact that these clients were introduced to a novel cleaner may have contributed to further stimulation of the DA system, considering that the response to novelty is also modulated by the DA system^49^. For instance, DA levels have also been reported to show a negative correlation in the zebrafish’s diencephalon in response to aggressive behaviour^53^. Furthermore, matrinxã (*Brycon amazonicus*) subjected to a social challenge (introduction of an intruder to their territory) showed lower hypothalamic DOPAC/DA ratios during fights^54^.

Clients exposed to a cleaner had higher DOPAC levels in the brain stem than clients exposed to a conspecific, which may be linked to the actual pursuit of cleaners and to all the coordination movements that these cleaning interactions require, such as getting close to cleaners and display-solicit (known as posing) for cleaners’ attention and then respond to cleaners’ service variations (to jolt and leave if necessary). Indeed, the mesencephalic locomotor region (MLR), an area of the brain stem, plays a crucial role in locomotor control: stimulation of the MLR elicits an increase of movement^63^.

The lower forebrain HVA levels of clients interacting with a cleaner, showed the opposite trend observed for DA levels, and may be explained by a reduction dopaminergic activity, which would be a consequence of a lower-release of HVA. On the other hand, the intensity of the conspecific challenge seems to have demanded of clients a boost of neurotransmission signalling, potentially contributing for an improvement of cognitive performance. For instance, treatment with *Olea europaea* oil increases HVA concentration in rats^64^, inducing exploratory activity and enhancing learning and memory^65^. It is perhaps the slowing down of the DA neural pathways (increase of DA and DOPAC production, decrease of HVA levels) at the diencephalon and brain stem of clients introduced to a cleaner that functions as a pre-requisite for cleaning to occur, providing to clients the physiological background that creates the motivation to seek to be cleaned. Clients’ perception of benefit is then further achieved with a reduction of their stress response^52^. The relationship between clients’ more specific behaviour and their brain 5-HIAA/5-HT and DOPAC/DA ratios, across studied regions, did not reveal any significant differences.

**Figure 3:**
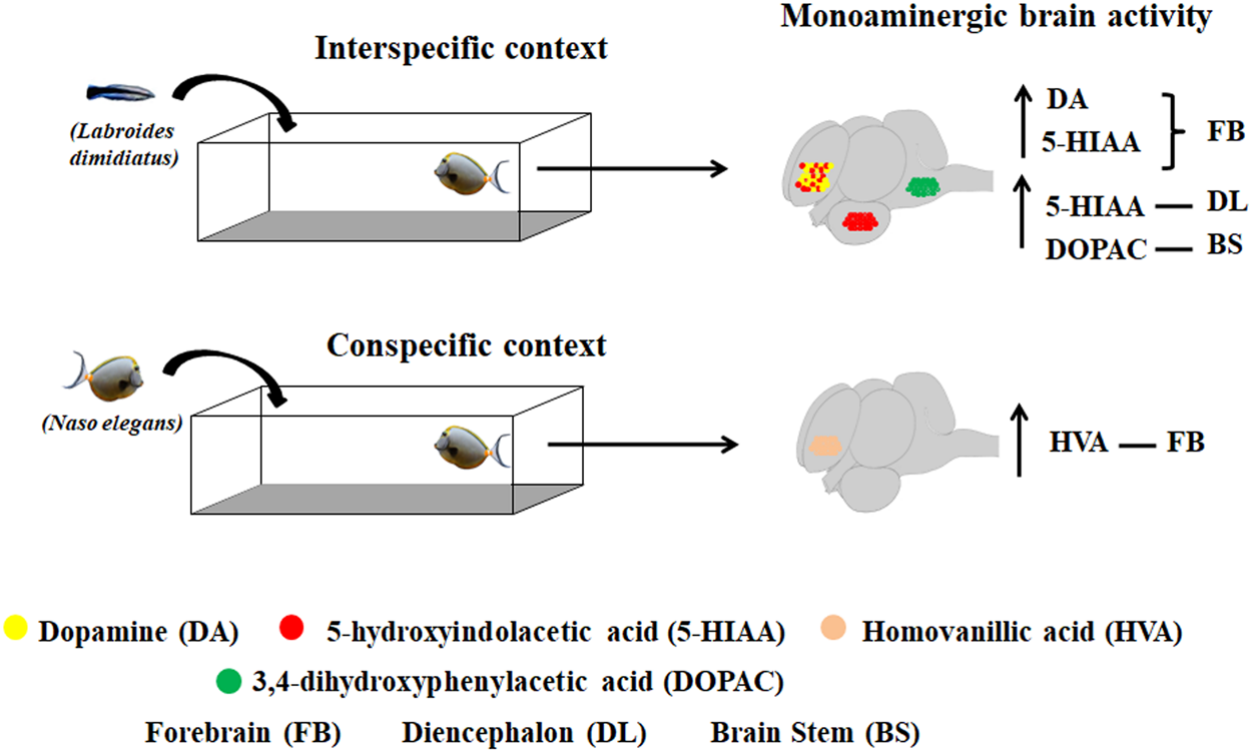
Monoaminergic brain activity in interspecific and conspecific contexts. *Labroides dimidiatus* and *Naso elegans* partial images were adapted from photos taken by MCS.

Overall, we demonstrate that monoaminergic activity differences occur at two main brain regions, the diencephalon and the forebrain of fish clients engaging in social and mutualistic activities (Fig. 3). The generally higher forebrain and diencephalic function, in the case of 5-HIAA and DA levels, as well as higher brain stem DOPAC levels and lower HVA in the diencephalon occur solely when clients are in contact with cleaners and not during conspecific engagement.

## Methods

### Animals and housing

Experiments were conducted at the fish housing facilities of the Oceanário de Lisboa (Lisbon, Portugal). The specimens used in this study were adult blond naso tang *N. elegans* (family Acanthuridae, aka clients), and the Indo-Pacific bluestreak cleaner wrasse *L. dimidiatus*, all imported to Portugal by a local distributor (Tropical Marine centre, Lisbon, Portugal). Total length (TL) and total weight (TW) of tang *N. elegans* ranged from 8.4 to 15.5 cm (mean ± SD: 11.6 ± 1.93 cm) and 10.21 to 49.61 g (25.22 ± 12.44 g). Tangs were kept in stock aquaria of 100 × 40 × 40 cm and cleaner wrasses were kept alone in 50 × 40 × 40 cm aquaria. All aquaria were combined in a flow through system that pumped water from a larger sump (150 × 50 × 40 cm) that served as a mechanical and biological filter. Nitrite concentration was kept to very low levels (always below 0.3 mg/l). Each tank contained an air supply and a commercial aquarium heater (125 W, Eheim, Jäger). PVC pipes (15–20 cm long; 20 cm diameter) served as shelter for the fish. Experiments were carried out in the individual smaller tanks (50 × 40 × 40 cm). The methods protocol was carried out in accordance to the approved guidelines by the Oceanário de Lisboa (fish housing facilities), where the experiments were then developed. Animal procedures used in this study were also approved by the Portuguese Veterinary Office (Direcção Geral de Veterinária, license #0420/000/000/2009).

### Experimental design and sampling

Our subject clients (*N. elegans*) were allocated to one of two treatment groups: (A) sympatric cleaner (*L. dimidiatus*) or (B) conspecific (*N. elegans*); n = 7 each. The focal clients were housed in the experimental tank for a minimum of two days before the stimulus client or cleaner fish was carefully introduced to the tank. Experimental aquaria were also divided by opaque partitions that prevented subject client fish from observing other individuals (inside other aquaria) during experiments. Behaviour was then videotaped for the next 60 minutes while the experimenter left the room (see section behavioural analyses below). At the end of experiments, each tang was rapidly captured and sacrificed with an overdose of tricaine solution, a powerful anesthetic (MS222, Pharmaq; 1000 mg/L) and the spinal cord sectioned (both methods aimed to reduce fish suffering). The brain was immediately dissected under a stereoscope (Zeiss; Stemi 2000) into four macro-areas: forebrain (includes olfactive bulbs and telencephalon), diencephalon, optic tectum and the brain stem. The cerebellum was also collected but was not included in the analysis due to a laboratorial incident which ruined the samples. Brain macroareas were stored at −80 °C.

Quantification of monoamines by high performance liquid chromatography with electrochemical detection (HPLC-EC).

The macroareas were homogenized in 4% (w/v) ice-cold perchloric acid containing 100 ng/ml 3,4-dihydroxybenzylamine (DHBA, the internal standard) using a Sonifier cell disruptor B-30 (Branson Ultrasonics, Danbury, CT, USA) and were immediately placed on dry ice. Subsequently, the homogenized samples were thawed and centrifuged at 21,000 × g for 10 min at 4 °C. The supernatant was used for high performance liquid chromatography with electrochemical detection (HPLC-EC), analyzing the monoamines dopamine (DA) and serotonin (5-HT, 5-hydroxytryptamine) the DA metabolite DOPAC (3,4-dihydroxyphenylacetic acid), 5-HT metabolite 5-HIAA (5-hydroxyindoleacetic acid), and homovanillic acid (HVA), as described by Overli *et al*.^66^. In brief, the HPLC–EC system consisted of a solvent delivery assystem model 582 (ESA, Bedford, MA, USA), an auto injector Midas type 830 (SparkHolland, Emmen, the Netherlands), a reverse phase column (Reprosil-Pur C18-AQ 3 µm, 100 mm × 4 mm column, Dr. Maisch HPLC GmbH, Ammerbuch-Entringen,Germany) kept at 40 °C and an ESA 5200 Coulochem II EC detector (ESA, Bedford, MA, USA) with two electrodes at reducing and oxidizing potentials of −40 mV and +320 mV. A guarding electrode with a potential of +450 mV was employed before the analytical electrodes to oxidize any contaminants. The mobile phase consisted of 75 mM sodium phosphate, 1.4 mM sodium octyl sulphate and 10 µM EDTA indeionized water containing 7% acetonitrile brought to pH 3.1 with phosphoric acid. Samples were quantified by comparison with standard solutions of known concentrations. To correct for recovery DHBA was used as an internal standard using HPLC software ClarityTM (DataApex Ltd., Prague, Czech Republic). The ratios of 5-HIAA/5-HT and DOPAC/DA were calculated and used as an index of serotonergic and dopaminergic activity, respectively. For normalization of brain monoamine levels, brain protein levels were determined with Bicinchoninic acid protein determination (Sigma–Aldrich, Sweden) according to the manufacturer’s instructions. The assay was read on Labsystems multiskan 352 plate reader (Labsystems, Thermo Fisher Scientific) wavelength of 570 nm.

### Behavioural analyses

During each video analysis, we recorded: (a) the number and duration (in seconds) of a cleaning inspection toward each client or cleaner fish, (b) the frequency and duration of tactile stimulation provided (where a cleaner touches, with fins, the body of the client and no feeding is involved^67^); (c) the number of jolts by clients (cleaners sometimes take bites to which the clients respond with a short body jolt that usually is a behaviour associated with cheating by cleaner fish^68,69^; (d) number and duration of chases where the subject (focal individual) rapidly advanced toward the other fish (in seconds); and finally (e) number of bites. In the conspecific context, although we tried to match the sizes of the individuals, this was not always possible. Thus, the incidence of chases by the subject may be due to size differences (aka intruder is larger than the resident or vice-versa), which we could not control for.

### Statistical analyses

Data were analysed using non-parametric tests because the assumptions for parametric testing were not met. Mann-Whitney U tests were performed to detect differences between treatments (two groups: client with a cleaner, and client with a conspecific) for each brain area and behavioural measures. The effect size was calculated using eta squared (η2) for Cohen’s d was used for U test comparisons. Finally, relationships within and between behavioural measures and clients brain monoaminergic levels were examined by using Spearman correlation coefficients. We proceed to correct our p values by applying the Benjamini-Hochberg false discovery rate correction^70^ reporting in the text just the correlations that remained significant. In Tables 2 and 3 all p values are reported.
